# The Next Generation of Clinical Studies with Antidepressants in Bipolar Disorder

**DOI:** 10.3389/fpsyt.2014.00027

**Published:** 2014-03-24

**Authors:** Rodrigo Machado-Vieira

**Affiliations:** ^1^LIM-27, Institute and Department of Psychiatry, University of Sao Paulo, Sao Paulo, Brazil; ^2^National Institute of Mental Health, National Institutes of Health, Bethesda, MD, USA

**Keywords:** bipolar disorder, depression, treatment, antidepressants, biomarkers

Antidepressants are still widely used in the treatment for bipolar disorder (BD), but evidence do not support their extensive use during depressive episodes. Recently, an important systematic review was published after an extensive literature search on the use of antidepressants in bipolar depression (1). Based on the conclusions, in spite of a considerable number of clinical studies (more than 37 so far) and meta-analyses currently available, the authors did not make broad statements endorsing antidepressant use in BD, but acknowledge that individual bipolar patients may benefit from these agents. Other studies showed similar results (2).

These publications also call attention to a wide range of methodological issues and discrepancies as well as to heterogeneous statistical approaches and conclusions. Also, studies have emphasized other limitations (2) including small samples, co-treatments, lack of adequate controls, and varied design, and therefore, current clinical evidence seems still inconclusive.

Thus, the first generation of studies has not provided consistent clinical answers to ensure more generalizable conclusions and guidelines for clinicians. They focused primarily in answering two simple questions: first, testing “yes or no” for the use of antidepressants regarding efficacy and safety (switch risk) in bipolar depression. This linear paradigm still does not help to guide clinicians’ therapeutic decision. The second question aimed to address what agent (or class) would be more effective and safer in bipolar depression. These two main aspects still remain not fully elucidated and additional questions have arisen, described next. Also, the role of BD diagnosis (bipolar I or II), family history and the risk of cycle acceleration, suicidality, mixed episodes/rapid cycling as associated outcomes did not provide yet consistent guidance. These variables were evaluated as secondary analyses and did not present standardized definitions (e.g., switch process and cycle acceleration). In the same perspective, even though it is clear that antidepressant monotherapy is contraindicated in bipolar depression, some large studies used this approach to compare with new agents (e.g., EMBOLDEN II).

Thus, this is an area where future studies critically require translational approach for the next generation of clinical trials (3). Only few trials with antidepressants in bipolar depression have systematically evaluated the potential clinical usefulness of biomarkers that may predict antidepressant response in BD and even less have thoroughly assessed the potential biological basis of switch risk. Most of the studies also have limitations including small samples, co-treatments, lack of adequate controls, and varied design.

The identification of neurobiological substrates able to minimize sample heterogeneity may also help to identify potential enriched samples for further confirmatory studies with different classes of antidepressants would be a critical step forward. This may also provide evidence to support the use of antidepressants in specific subgroups and guide clinical decision in the personalized treatment paradigm. At the same time, the development of standardized conceptual definitions is essential to perform neurobiological studies and may be added to the evaluation of diverse constructs and dimensions across neurobiological systems.

In the same context, the next generation of proof of concept studies is also expected to provide information on which clinical and neurobiological variables are associated with a subgroup that will likely respond to a specific antidepressant and address key questions including optimal duration of antidepressant treatment and presence (or not) of a dose- and/or time-dependent regulation of mood elevation using adequate methodology (e.g., more frequent rating scales, standard dose escalation design). These approaches may also help to define the lowest effective dose for the shortest time necessary for sustained remission. Future studies would also benefit from dimensional approaches and evaluation of new targets such as the glutamatergic system.

Finally, it is also critical to better understand this complex and non-linear interaction, the study of specific domains such as early improvement, short and long-term response, cognition, role of compliance, duration of use, and dosage/response curve. These are important variables to be considered integrated with biomarkers such as predictors of response and surrogate outcomes. The next generation of studies is also expected to evaluate a broader risk–benefit equation, integrating generalizable data to discoveries on personalized treatments for those who need it most.
